# Gluten intake and metabolic health: conflicting findings from the UK Biobank

**DOI:** 10.1007/s00394-020-02351-9

**Published:** 2020-08-06

**Authors:** Inken Behrendt, Mathias Fasshauer, Gerrit Eichner

**Affiliations:** 1 Institute of Nutritional Science, Justus-Liebig-University of Giessen, Goethestr. 55, 35390 Giessen, Germany; 2 Department of Internal Medicine (Endocrinology, Nephrology, and Rheumatology), University of Leipzig, Leipzig, Germany; 3 Leipzig University Medical Center, IFB AdiposityDiseases, Leipzig, Germany; 4 Mathematical Institute, Justus-Liebig-University of Giessen, Giessen, Germany

**Keywords:** Body composition, Dyslipidemia, Gluten, Hypertension, Metabolic health, Obesity

## Abstract

**Purpose:**

The impact of gluten intake on metabolic health in subjects without celiac disease is unclear. The present study aimed to assess the association between gluten intake and body fat percentage (primary objective), as well as a broad set of metabolic health markers.

**Methods:**

Gluten intake was estimated in 39,927 participants of the UK Biobank who completed a dietary questionnaire for assessment of previous 24-h dietary intakes. Multiple linear regression analyses were performed between gluten intake and markers of metabolic health with Holm adjustment for multiple comparisons.

**Results:**

Median gluten intake was 9.7 g/day (male: 11.7 g/day; female: 8.2 g/day; *p* < 0.0001). In multiple linear regression analysis, association between gluten intake and percentage body fat was negative in males (*β* = − 0.028, *p* = 0.0020) and positive in females (*β* = 0.025, *p* = 0.0028). Furthermore, gluten intake was a negative predictor of total cholesterol (male: *β* = − 0.031, *p* = 0.0154; female: *β* = − 0.050, *p* < 0.0001), high-density lipoprotein cholesterol (male: *β* = − 0.052, *p* < 0.0001; female: *β* = − 0.068, *p* < 0.0001), and glomerular filtration rate (sexes combined: *β* = − 0.031, *p* < 0.0001) in both sexes. In females only, gluten intake was positively associated with waist circumference (*β* = 0.041, *p* < 0.0001), waist-to-height ratio (*β* = 0.040, *p* < 0.0001), as well as body mass index (*β* = 0.043, *p* < 0.0001), and negatively related to low-density lipoprotein cholesterol (*β* = − 0.035, *p* = 0.0011). A positive association between gluten intake and triglycerides was observed in males only (*β* = 0.043, *p* = 0.0001).

**Conclusion:**

This study indicates that gluten intake is associated with markers of metabolic health. However, all associations are weak and not clinically meaningful. Limiting gluten intake is unlikely to provide metabolic health benefits for a population in total.

**Electronic supplementary material:**

The online version of this article (10.1007/s00394-020-02351-9) contains supplementary material, which is available to authorized users.

## Introduction

Gluten, the major storage protein of wheat, is a complex protein structure consisting of monomeric prolamins and polymeric glutenins which are also present in other cereals such as rye and barley [1]. Gluten is partly resistant to intestinal digestion, resulting in the formation of immunogenic oligopeptides some of which are capable of triggering celiac disease (CD) in genetically susceptible individuals which is an autoimmune-mediated disorder [2]. Gluten ingestion is also considered to trigger further autoimmune diseases such as diabetes mellitus type 1 [3]. It is indisputable that the only effective treatment for CD is lifelong adherence to a strict gluten-free diet (GFD) [2] but gluten-free and gluten-limited diets also gained in popularity in healthy people [4]. The prevalence of CD in Western populations is only about 1% of the general population [5]. In contrast, 3.7% of the general UK population are avoiding gluten [6]. In addition, data from the National Health and Nutrition Examination Survey (NHANES) revealed that the prevalence of people on a GFD rose from 0.5% in 2009–2010 to 1.7% in 2013–2014 [7].

Dietary gluten has been linked to adverse health outcomes such as obesity, metabolic syndrome, and cardiovascular risk independent of CD in the lay public [8, 9]. Many patients consider a GFD as balanced, healthy, and useful for weight control due to its restrictive nature [10]. Adverse metabolic effects of gluten are supported by animal experiments. Thus, addition of 4.5% of wheat gluten to a normal chow and a high-fat diet increased body weight and fat deposits without changing food intake and lipid excretion in male C57BL/6 mice [11, 12]. In another study, mice fed a defined high-fat diet containing 4% gliadin displayed higher glycated hemoglobin A1c (HbA1c), higher insulin resistance, and more hepatic lipid accumulation [13].

In contrast to these animal studies, evidence for a beneficial role of gluten also exists. Thus, a GFD may result in limited food choice and an unbalanced diet which is low in B vitamins, micronutrients, and fiber intake [14]. Moreover, a GFD might increase the risk for metabolic syndrome in CD patients [10, 15]. Improved intestinal absorption, as well as the high content of sugar, fat, and energy in gluten-free products might contribute [10]. A recent systematic review concluded that a GFD adversely affects cardiovascular risk factors in people suffering from CD including increases in body mass index (BMI), total cholesterol, and fasting blood glucose [16]. Furthermore, high gluten intake has been linked to improved metabolic and vascular health. Dietary gluten intake was inversely associated with diabetes mellitus type 2 risk among healthy people in three large prospective US studies [17]. In contrast, gluten intake was not significantly associated with risk of coronary artery disease in a similar study population [18].

Taking published evidence into consideration, the impact of gluten intake on metabolic health remains controversial. Furthermore, both depth of metabolic characterization and sample size in human studies on gluten and metabolism have been limited so far. To address these limitations, in the present study, the association between gluten intake and a broad set of markers of metabolic health is elucidated in a large, well-characterized population of 39,927 UK Biobank participants. We hypothesized that gluten intake is negatively associated with metabolic health after adjusting for confounders.

## Methods

### Study and participants

This study was conducted with data from the UK Biobank which is a large ongoing prospective cohort study not representative of the general UK population [19]. The UK Biobank study aims to improve the prevention, diagnosis, and treatment of serious public health risks like metabolic syndrome and cardiovascular disease [19]. In brief, more than 500,000 participants were recruited from across the UK at 22 assessment centers between 2006 and 2010. UK Biobank obtained ethical approval for this study by the North West Multicenter Research Ethics Committee and all participants gave written informed consent to participate and be followed up [19].

### Demographics

At the baseline assessment, sociodemographic characteristics including sex, age, ethnic background, qualifications, and average total household income per year, as well as lifestyle risk factors including smoking status and physical activity, were self-reported and collected using a touchscreen questionnaire [19].

### Medical history

In a verbal interview, participants reported trained staff members on diagnoses of previous and current medical conditions, as well as on prescription medications [20].

### Physical examination

Physical measurements were undertaken by trained staff members using standard operating procedures. Systolic blood pressure (SBP) and diastolic blood pressure (DBP) were measured with the Omron 705 IT electronic blood pressure monitor (OMRON Healthcare Europe B.V. Hoofddorp, The Netherlands). Two blood pressure measurements were taken with a resting period of at least 1 min [21].

### Anthropometry and body composition

Standing height was measured barefoot to the nearest centimeter using a Seca 240-cm height measure (Seca GmbH & Co. KG, Hamburg, Germany) [22]. Hip circumference (HC) and waist circumference (WC) were measured to the nearest cm using a Seca 200-cm tape measure (seca GmbH & Co. KG, Hamburg, Germany). Waist measurement was recorded at the smallest part of the trunk on the outbreath. In cases where a natural indent could not be found, the tape was passed around the level of the umbilicus. HC was obtained at the widest part of the hips [22]. The measurements were later used to calculate waist-to-hip ratio (WHR) and waist-to-height ratio (WHtR). Body weight and whole body fat mass were measured with a Tanita BC418MA body composition analyzer (Tanita Europe B.V., Amsterdam, The Netherlands) to the nearest 0.1 kg by bioelectrical impedance. Body mass index was calculated as weight in kg divided by height in m^2^ [22].

### Dietary assessment and estimation of gluten intake

To provide more detailed dietary information, a web-based dietary questionnaire for assessment of previous 24-h dietary intakes (Oxford WebQ) was completed by 211,013 participants [23]. The Oxford WebQ was specifically developed for use in large population studies and has been validated against an interviewer-administered 24-h dietary recall [23]. A subgroup of 70,710 participants conducted the Oxford WebQ at the assessment center during their baseline visit. Gluten intake within our study was estimated exclusively in these 70,710 participants since all clinical and dietary assessments were performed at the same time point. The following exclusion criteria were applied similar to Anderson and co-workers [24]: implausible energy intake (overall energy intake < 1.1 × basal metabolic rate, overall energy intake > 18,828 kJ), outlier values (BMI < 14.9 kg/m^2^ or > 60 kg/m^2^; WC or percentage body fat > 4 standard deviations from mean values). Participants with self-reported history of malabsorption/CD and cancer, suspected or confirmed pregnancy, as well as on a low-calorie diet, were also excluded resulting in a study population of 39,927 participants (Online Resource 1).

Assessment of gluten intake was based on the Oxford WebQ. All food items containing wheat, rye, or barley were considered as containing gluten. Overall, 44 of the 230 dietary items in the Oxford WebQ were regarded as gluten-containing. For food categories containing multiple food items (e.g., bread roll, bap, burger bun, hotdog roll, bagel), the gluten content of all subitems was summed up and divided by the total number of subitems similar to [25]. Trace amounts of gluten which are included in processed food were not estimated similar to [17] since contribution to total gluten intake would be negligible. There is no detailed information about gluten content of food products in any food database or the literature. In accordance with recent studies, gluten content was estimated by multiplying cereal protein intake by 0.75 [17, 18]. Cereal protein content for each item was determined using McCance and Widdowson’s The Composition of Foods and its supplements on which all calculations of energy and nutrient intake of the Oxford WebQ are based [23]. In addition, product labels and recipes from cookbooks were used for the estimation of cereal protein content. In the Oxford WebQ, participants indicate the number of standard portions consumed of specific food items. For each participant, average daily gluten intake (g/day) was calculated by multiplying the frequency of each gluten-containing item by the estimated gluten content of that particular item in a standard portion. Standard portion sizes were taken from food portion sizes [26] as suggested by the developers of the Oxford WebQ [23]. For food items not listed in [26], product labels were used. Energy-adjusted gluten intake (mg kJ^−1^ d^−1^) was calculated by dividing average daily gluten intake (g/day) by daily energy intake (kJ) provided by the Oxford WebQ and multiplying by 1000.

### Metabolic profile

Non-fasting venous blood samples (~ 50 ml) were drawn by trained staff members at the assessment centers. All samples were analyzed at the central laboratory in Stockport. Serum biomarker analyses were performed using clinical chemistry analyzers (Beckman Coulter AU5800; Beckman Coulter, Brea, CA, USA, and Siemens Advia 18,000, Siemens Healthineers, Erlangen, Germany). Assay methods, reagent suppliers, reportable ranges, and assay quality procedures are available on the UK Biobank website [27, 28]. HbA1c was measured in packed red blood cells using a Bio-Rad Variant II Turbo Haemoglobin Testing System (Bio-Rad Laboratories, Inc., Hercules, CA, USA) [29]. Glomerular filtration rate (GFR) was calculated using the combined creatinine–cystatin C equation described in [30].

### Physical activity

Assessment of physical activity has been described elsewhere [31]. In brief, time spent in walking, moderate, and vigorous activity was weighted by the energy expended for these categories of activity referred by the International Physical Activity Questionnaire [31]. Total physical activity was measured as metabolic equivalent task [MET]-min/week.

### Statistical analyses

Data were analyzed using R software version 3.6.1 [32] together with the add-on packages tidyverse [33], nephro [34], readxl [35], venn [36], car [37], effects [38], skimr [39], and lm.beta [40]. All multiple linear regression models contained as independent covariates sex, age, ethnic background, qualifications, average total household income per year, smoking status, and physical activity to adjust for their influence on the respective dependent variable. Further adjustments for specific dependent variables are summarized in Table 2. Continuous independent variables with a heavily skewed distribution were lg_10_ transformed. Holm adjustment was used to control for multiple comparisons. A *p *value of < 0.05 was considered as statistically significant.

## Results

### Gluten intake and energy-adjusted gluten intake in UK Biobank participants

Baseline characteristics of the study population are summarized in Table 1. Median (Q1–Q3) age of the study population was 57 (49–63) with 55.3% of participants being female. Median gluten intake in UK Biobank participants was 9.7 (6.2–13.7) g/day. Median energy-adjusted gluten intake was 1.00 (0.67–1.38) mg kJ^−1^ d^−1^ (Table 1). Gluten intake was higher in male [11.7 (8.1–15.9) g/day] as compared to female [8.2 (5.2–11.6) g/day] participants (*p *< 0.0001; Online Resource 2a). Similarly, energy-adjusted gluten intake was higher in male subjects [male: 1.10 (0.77–1.47) mg kJ^−1^ d^−1^; female: 0.93 (0.61–1.30) mg kJ^−1^ d^−1^; *p* < 0.0001; Online Resource 2b]. Gluten intake was positively correlated with total energy intake in univariate analysis (Pearson’s r = 0.41, *p* < 0.0001).

**Table 1:** Baseline characteristics of the UK Biobank cohort by quartiles of gluten intake (g/day) and sex

Characteristics	All (*n*=39,927)	Male (*n*=17,851)				Female (*n*=22,076)			
		Quartile 1	Quartile 2	Quartile 3	Quartile 4	Quartile 1	Quartile 2	Quartile 3	Quartile 4
Gluten intake (g/d)	9.7 (6.2–13.7)	5.8 (4.1–7.0)	10.0 (9.1–10.8)	13.7 (12.7–14.7)	19.2 (17.4–22.5)	3.4 (1.8–4.7)	6.7 (6.0–7.4)	9.8 (9.0–10.6)	14.5 (12.8–17.0)
Energy-adjusted gluten intake (mg * kJ^-1^ * d^-1^)	1.00 (0.67–1.38)	0.57 (0.40–0.71)	0.98 (0.84–1.12)	1.28 (1.10–1.47)	1.72 (1.44–2.03)	0.40 (0.21–0.56)	0.79 (0.66–0.92)	1.12 (0.95–1.30)	1.56 (1.31–1.86)
Gluten-free diet	628 (1.6)	67 (1.5)	25 (0.6)	20 (0.4)	22 (0.5)	299 (5.4)	102 (1.8)	50 (0.9)	43 (0.8)
Age (years)	57 (49–63)	60 (51–64)	59 (51–64)	58 (50–63)	56 (48–62)	58 (50–63)	58 (50–63)	56 (49–62)	55 (47–62)
Smoking status									
Never	22,992 (57.6)	2250 (50.4)	2244 (50.3)	2299 (51.5)	2376 (53.2)	3353 (60.8)	3433 (62.2)	3495 (63.3)	3542 (64.2)
Previous	13,532 (33.9)	1781 (39.9)	1746 (39.1)	1700 (38.1)	1608 (36.0)	1777 (32.2)	1686 (30.5)	1645 (29.8)	1589 (28.8)
Current	3313 (8.3)	425 (9.5)	461 (10.3)	455 (10.2)	66 (10.4)	76 (6.8)	386 (7.0)	367 (6.6)	377 (6.8)
Pnta	90 (0.2)	7 (0.2)	12 (0.3)	9 (0.2)	12 (0.3)	13 (0.2)	14 (0.3)	12 (0.2)	11 (0.2)
Ethnic background									
White	37,718 (94.5)	4179 (93.6)	4285 (96.0)	4305 (96.5)	4223 (94.6)	5115 (92.7)	5205 (94.3)	5220 (94.6)	5186 (94.0)
Black	554 (1.4)	64 (1.4)	40 (0.9)	26 (0.6)	51 (1.1)	113 (2.0)	91 (1.6)	78 (1.4)	91 (1.6)
Asian	893 (2.2)	133 (3.0)	71 (1.6)	68 (1.5)	114 (2.6)	157 (2.8)	108 (2.0)	102 (1.8)	140 (2.5)
Mixed	296 (0.7)	32 (0.7)	25 (0.6)	19 (0.4)	27 (0.6)	54 (1.0)	50 (0.9)	46 (0.8)	43 (0.8)
Other	306 (0.8)	28 (0.6)	17 (0.4)	28 (0.6)	28 (0.6)	60 (1.1)	44 (0.8)	55 (1.0)	46 (0.8)
Pnta or Dnk	160 (0.4)	27 (0.6)	25 (0.6)	17 (0.4)	19 (0.4)	20 (0.4)	21 (0.4)	18 (0.3)	13 (0.2)
Qualifications									
Noa	3444 (8.6)	392 (8.8)	387 (8.7)	423 (9.5)	417 (9.3)	415 (7.5)	469 (8.5)	476 (8.6)	465 (8.4)
Other	1924 (4.8)	178 (4.0)	189 (4.2)	175 (3.9)	168 (3.8)	329 (6.0)	296 (5.4)	320 (5.8)	269 (4.9)
NVQ or HND or HNC equivalent	2067 (5.2)	330 (7.4)	340 (7.6)	328 (7.3)	346 (7.8)	165 (3.0)	160 (2.9)	189 (3.4)	209 (3.8)
CSEs or equivalent	1843 (4.6)	164 (3.7)	193 (4.3)	233 (5.2)	257 (5.8)	233 (4.2)	228 (4.1)	246 (4.5)	289 (5.2)
O levels/GCSEs or equivalent	8515 (21.3)	790 (17.7)	815 (18.3)	872 (19.5)	905 (20.3)	1266 (22.9)	1323 (24.0)	1287 (23.3)	1257 (22.8)
A levels/AS levels or equivalent	5267 (13.2)	527 (11.8)	552 (12.4)	526 (11.8)	533 (11.9)	817 (14.8)	777 (14.1)	775 (14.0)	760 (13.8)
College or university degree	16,640 (41.7)	2053 (46.0)	1961 (43.9)	1882 (42.2)	1811 (40.6)	2260 (40.9)	2235 (40.5)	2195 (39.8)	2243 (40.6)
Pnta	227 (0.6)	29 (0.6)	26 (0.6)	24 (0.5)	25 (0.6)	34 (0.6)	31 (0.6)	31 (0.6)	27 (0.5)
Total household income per year (£)									
<18000	5978 (15.0)	548 (12.3)	596 (13.4)	647 (14.5)	656 (14.7)	768 (13.9)	891 (16.1)	876 (15.9)	996 (18.0)
18000–30999	8802 (22.0)	940 (21.1)	981 (22.0)	987 (22.1)	981 (22.0)	1190 (21.6)	1269 (23.0)	1243 (22.5)	1211 (21.9)
31000–51999	9897 (24.8)	1170 (26.2)	1231 (27.6)	1192 (26.7)	1189 (26.6)	1286 (23.3)	1274 (23.1)	1282 (23.2)	1273 (23.1)
52000–100000	8287 (20.8)	1056 (23.7)	1012 (22.7)	1016 (22.8)	1016 (22.8)	1095 (19.8)	1033 (18.7)	1020 (18.5)	1039 (18.8)
>100000	2608 (6.5)	397 (8.9)	319 (7.1)	283 (6.3)	267 (6.0)	385 (7.0)	309 (5.6)	328 (5.9)	320 (5.8)
Pnta or Dnk	4355 (10.9)	352 (7.9)	324 (7.3)	338 (7.6)	353 (7.9)	795 (14.4)	743 (13.5)	770 (14.0)	680 (12.3)
Energy intake									
kJ/d	9452 (8058–11207)	9673 (8573–11048)	10101 (8945–11635)	10650 (9312–12322)	11692 (10070–13688)	7877 (6917–9127)	8348 (7308–9725)	8719 (7582–10248)	9597 (8225–11358)
kcal/d	2259 (1926–2678)	2312 (2049–2641)	2414 (2138–2781)	2546 (2226–2945)	2794 (2407–3272)	1883 (1653–2181)	1995 (1747–2324)	2084 (1812–2449)	2294 (1966–2715)
Carbohydrate intake (%Energy)	48.2 (42.5–53.8)	45.8 (39.2–51.7)	46.4 (40.5–52.2)	47.5 (42.1–52.8)	49.6 (44.3–54.7)	47.3 (40.9–53.7)	48.7 (42.9–54.2)	48.7 (43.6–53.9)	50.5 (45.3–55.6)
Protein intake (%Energy)	14.8 (12.6–17.2)	14.7 (12.5–17.3)	14.5 (12.5–16.8)	14.2 (12.2–16.3)	14.0 (12.1–16.2)	15.8 (13.3–18.5)	15.2 (12.8–17.6)	14.9 (12.7–17.4)	14.7 (12.5–17.1)
Fat intake (%Energy)	31.7 (26.8–36.6)	31.4 (26.3–36.5)	31.9 (27.3–36.9)	31.7 (27.0–36.3)	31.0 (26.2–35.6)	31.6 (26.2–37.1)	32.0 (27.1–36.9)	32.5 (27.6–37.2)	31.5 (26.6–36.3)
Fiber intake (g/d)	17.1 (13.0–22.0)	15.6 (11.4–20.0)	16.5 (12.5–21.3)	18.4 (14.0–23.1)	20.8 (16.1–26.4)	15.1 (11.3–19.6)	15.9 (12.2–20.3)	16.9 (13.1–21.4)	19.0 (14.8–24.1)
Total physical activity (MET-min/week)	1911 (924–3672)	1866 (933–3653)	1884 (874–3652)	1926 (924–3756)	2026 (956–4040)	1977 (956–3672)	1890 (919–3546)	1893 (914–3546)	1866 (876–3590)
Percentage body fat	29.8 (24.1–36.3)	24.2 (20.5–27.6)	24.1 (20.5–27.6)	24.3 (20.6–27.9)	24.2 (20.2–28.0)	34.8 (30.2–39.2)	35.3 (30.7–39.8)	35.6 (30.8–40.1)	35.7 (30.8–40.7)
WC (cm)	87 (78–96)	93 (87–100)	93 (87–100)	94 (88–101)	95 (88–102)	79 (73–87)	80 (74–88)	81 (74–89)	82 (74–91)
WHR	0.86 (0.79–0.93)	0.92 (0.88–0.96)	0.92 (0.88–0.96)	0.93 (0.89–0.97)	0.92 (0.88–0.97)	0.80 (0.76–0.85)	0.80 (0.76–0.85)	0.80 (0.76–0.86)	0.81 (0.76–0.86)
WHtR	0.51 (0.47–0.56)	0.53 (0.49–0.57)	0.53 (0.49–0.57)	0.53 (0.50–0.57)	0.53 (0.49–0.58)	0.49 (0.45–0.54)	0.49 (0.45–0.54)	0.49 (0.45–0.55)	0.50 (0.45–0.56)
BMI (kg/m^2^)	25.7 (23.4–28.6)	26.2 (24.2–28.5)	26.3 (24.2–28.7)	26.6 (24.4–29.1)	26.8 (24.4–29.4)	24.7 (22.4–27.4)	24.9 (22.7–28.0)	25.2 (22.7–28.4)	25.5 (22.8–29.2)
SBP (mmHg)	137 (125–151)	140 (129–154)	140 (129–153)	140 (129–153)	139 (128–151)	135 (122–149)	134 (122–149)	134 (122–150)	134 (121–148)
DBP (mmHg)	81 (74–88)	83 (76–90)	83 (76–90)	83 (76–90)	82 (76–90)	79 (72–87)	79 (73–87)	79 (73–87)	79 (73–87)
HbA1c (mmol/mol)	35.0 (32.6–37.5)	35.0 (32.6–37.4)	35.0 (32.8–37.4)	35.0 (32.6–37.7)	35.0 (32.6–37.6)	34.9 (32.5–37.3)	35.1 (32.7–37.5)	35.1 (32.7–37.4)	35.1 (32.6–37.5)
Total cholesterol									
mmol/l	5.7 (5.0–6.4)	5.6 (4.9–6.3)	5.5 (4.8–6.3)	5.5 (4.8–6.2)	5.5 (4.8–6.2)	5.9 (5.2–6.7)	5.9 (5.2–6.7)	5.8 (5.1–6.5)	5.7 (5.0–6.5)
mg/dl	220 (192–249)	215 (188–242)	214 (187–243)	213 (184–241)	211 (184–239)	229 (201–257)	228 (201–258)	225 (199–253)	221 (194–250)
LDL cholesterol									
mmol/l	3.5 (3.0–4.1)	3.5 (3.0–4.1)	3.5 (2.9–4.1)	3.5 (2.9–4.1)	3.5 (2.9–4.0)	3.6 (3.0–4.2)	3.6 (3.1–4.2)	3.6 (3.0–4.1)	3.5 (3.0–4.1)
mg/dl	136 (115–159)	136 (114–157)	135 (114–158)	135 (113–157)	133 (112–156)	138 (117–161)	140 (118–162)	137 (117–159)	135 (114–159)
HDL cholesterol									
mmol/l	1.5 (1.2–1.7)	1.3 (1.1–1.5)	1.3 (1.1–1.5)	1.3 (1.1–1.5)	1.3 (1.1–1.5)	1.7 (1.4–1.9)	1.6 (1.4–1.9)	1.6 (1.4–1.9)	1.6 (1.3–1.8)
mg/dl	56 (47–67)	51 (44–60)	50 (43–59)	49 (42–58)	49 (42–57)	64 (55–75)	63 (54–73)	62 (53–72)	61 (52–71)
Triglyceride									
mmol/l	1.4 (1.0–2.0)	1.5 (1.1–2.2)	1.6 (1.1–2.3)	1.7 (1.2–2.4)	1.6 (1.1–2.4)	1.2 (0.9–1.7)	1.3 (0.9–1.8)	1.3 (0.9–1.8)	1.3 (0.9–1.9)
mg/dl	123 (87–178)	135 (96–196)	142 (99–203)	145 (101–211)	144 (100–208)	106 (78–148)	111 (81–155)	113 (82–159)	113 (83–162)
CRP (mg/dl)	1.1 (0.6–2.3)	1.1 (0.6–2.1)	1.1 (0.6–2.1)	1.1 (0.6–2.1)	1.1 (0.6–2.1)	1.1 (0.5–2.2)	1.1 (0.5–2.3)	1.2 (0.6–2.4)	1.2 (0.6–2.7)
GFR (ml/min)	106 (95–116)	99 (89–108)	99 (89–108)	99 (89–108)	100 (90–110)	112 (102–121)	111 (101–120)	111 (101–120)	113 (102–122)

Categorical variables are presented as number (percentage) and continuous variables as median (Q1–Q3). *BMI* body mass index, *CRP* C-reactive protein, *CSE* Certificate of Secondary Education, *DBP* diastolic blood pressure, *Dnk* do not know, *GCSE* General Certificate of Secondary Education, *GFR* glomerular filtration rate, *HbA1c* hemoglobin A1c, *HDL* high-density lipoprotein, *HNC* Higher National Certificate, *HND* Higher National Diploma, *LDL* low-density lipoprotein, *MET* metabolic equivalent of task, *Noa* none of the above, *NVQ* National Vocational Qualification *Pnta* prefer not to answer, *Q* quartile, *SBP* systolic blood pressure, *WC* waist circumference, *WHR* waist-to-hip ratio, *WHtR* waist-to-height ratio

### Gluten intake and percentage body fat

**Fig. 1:**
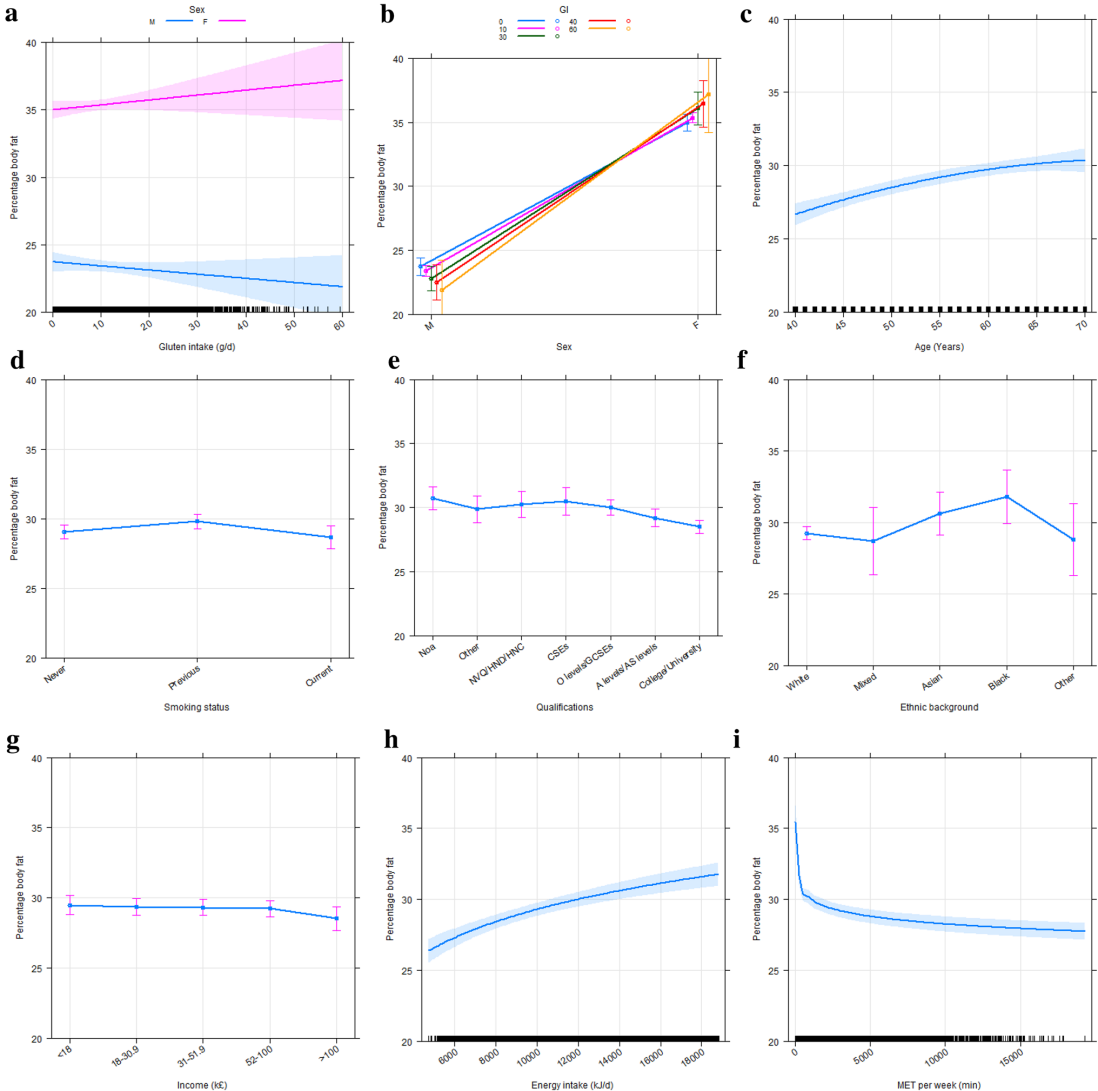
Predictor effects plots of multiple linear regression analysis determining association of percentage body fat (dependent variable) with **a** gluten intake, **b** sex, **c** age, **d** smoking status, **e** qualifications, **f** ethnic background, **g** income, **h** energy intake, and **i** MET per week (independent variables)

In multiple linear regression analysis, association between gluten intake and percentage body fat was negative in males (*β* = − 0.028, *p* = 0.0020) and positive in females (*β* = 0.025, *p* = 0.0028) after controlling for sex, age, smoking status, ethnic background, qualifications, total household income, energy intake, and physical activity (Table 2 and Fig. 1a). An increase of gluten intake by 1 g/day was associated with a decrease in percentage body fat by 0.03 percentage points in males and an increase by 0.04 percentage points in females (Table 2 and Fig. 1a). Among the covariates studied, major positive predictors of percentage body fat were female sex, increasing age, and energy intake, as well as decreasing physical activity (Fig. 1b, c, h, i). Similar findings were obtained for energy-adjusted gluten intake (Online Resource 3). Association between gluten intake and energy-adjusted gluten intake on the one hand and percentage body fat on the other hand was not altered in multiple linear regression models further adjusted for markers of diet quality, i.e. consumption of cooked vegetables, salad, fresh fruit, oily fish, processed meat, added salt, saturated fat, polyunsaturated fat, and fiber (Online Resource 4a and c). Further sensitivity analyses revealed that both gluten intake and energy-adjusted gluten intake remained associated with percentage body fat when participants with implausible energy intake were included in the multiple linear regression analysis (Online Resource 4b and d). Association between gluten intake and energy-adjusted gluten intake on the one hand and percentage body fat on the other hand remained similar if participants with diabetes mellitus, arterial hypertension, or dyslipidemia were excluded from analysis (Online Resource 5).

**Table 2:** Association between gluten intake (independent variable) and markers of metabolic health (dependent variable) depending on sex in multiple linear regression analyses

Dependent variable	*n*	Adjusted R^2^	Independent variable	*B*	*β*	SE	*t* value	Raw *p* value	Holm-adjusted *p* value
Percentage body fat	30,460	0.50	Gluten intake male	− 3.1 × 10^−2^	− 2.8 × 10^−2^	7.9 × 10^−3^	− 3.863	0.0001	0.0020
			Gluten intake female	3.6 × 10^−2^	2.5 × 10^−2^	9.6 × 10^−3^	3.769	0.0002	0.0028
Lg_10_ WC	30,460	0.33	Gluten intake male	− 6.7 × 10^−5^	− 8.2 × 10^−3^	6.9 × 10^−5^	− 0.976	0.3291	1.0000
			Gluten intake Female	4.5 × 10^−4^	4.1 × 10^−2^	8.3 × 10^−5^	5.346	<0.0001	<0.0001
Lg_10_ WHR	30,459	0.49	Gluten intake male	− 2.7 × 10^−5^	− 4.6 × 10^−3^	4.3 × 10^−5^	− 0.635	0.5257	1.0000
			Gluten intake female	6.2 × 10^−5^	8.0 × 10^−3^	5.2 × 10^−5^	1.202	0.2292	1.0000
Lg_10_ WHtR	30,460	0.17	Gluten intake male	− 1.1 × 10^−4^	− 1.4 × 10^−2^	7.0 × 10^−5^	− 1.534	0.1251	1.0000
			Gluten intake female	4.0 × 10^−4^	4.0 × 10^−2^	8.5 × 10^−5^	4.735	<0.0001	<0.0001
Lg_10_ BMI (kg/m²)	30,460	0.11	Gluten intake male	− 1.1 × 10^−4^	− 1.2 × 10^−2^	8.5 × 10^−5^	− 1.268	0.2049	1.0000
			Gluten intake female	5.0 × 10^−4^	4.3 × 10^−2^	1.0 × 10^−4^	4.872	<0.0001	<0.0001
Lg_10_ SBP (mmHg)	30,454	0.19	Gluten intake male	2.7 × 10^−5^	3.4 × 10^−3^	7.3 × 10^−5^	0.365	0.7151	1.0000
			Gluten intake female	− 7.9 × 10^−5^	− 7.5 × 10^−3^	8.8 × 10^−5^	− 0.890	0.3734	1.0000
Lg_10_ DBP (mmHg)	30,454	0.12	Gluten intake male	− 7.1 × 10^−5^	− 9.5 × 10^−3^	7.2 × 10^−5^	− 0.990	0.3220	1.0000
			Gluten intake female	− 2.1 × 10^−4^	− 2.1 × 10^−2^	8.7 × 10^−5^	− 2.426	0.0153	0.2136
HbA1c (mmol/mol)	28,371	0.35	Gluten intake male	1.0 × 10^−2^	1.4 × 10^−2^	6.4 × 10^−3^	1.606	0.1084	1.0000
			Gluten intake female	− 4.4 × 10^−3^	− 4.4 × 10^−3^	7.8 × 10^−3^	− 0.560	0.5757	1.0000
Lg_10_ total cholesterol (mmol/l)	28,740	0.21	Gluten intake male	− 3.5 × 10^−4^	− 3.1 × 10^−2^	1.1 × 10^−4^	− 3.302	0.0010	0.0154
			Gluten intake female	− 7.4 × 10^−4^	− 5.0 × 10^−2^	1.3 × 10^−4^	− 5.766	<0.0001	<0.0001
LDL cholesterol (mmol/l)	28,688	0.19	Gluten intake male	− 2.5 × 10^−3^	− 2.2 × 10^−2^	1.1 × 10^−3^	− 2.322	0.0203	0.2632
			Gluten intake female	− 5.1 × 10^−3^	− 3.5 × 10^−2^	1.3 × 10^−3^	− 4.023	<0.0001	0.0011
Lg_10_ HDL cholesterol (mmol/l)	26,184	0.31	Gluten intake male	− 7.5 × 10^−4^	− 5.2 × 10^−2^	1.4 × 10^−4^	− 5.588	<0.0001	<0.0001
			Gluten intake female	− 1.3 × 10^−3^	− 6.8 × 10^−2^	1.6 × 10^−4^	− 7.985	<0.0001	<0.0001
Lg_10_ triglyceride (mmol/l)	28,728	0.20	Gluten intake male	1.3 × 10^−3^	4.3 × 10^−2^	2.8 × 10^−4^	4.484	<0.0001	0.0001
			Gluten intake female	9.7 × 10^−4^	2.5 × 10^−2^	3.4 × 10^−4^	2.871	0.0041	0.0613
Lg_10_ CRP (mg/dl)	28,678	0.23	Gluten intake male	− 2.5 × 10^−4^	− 4.2 × 10^−3^	5.4 × 10^−4^	− 0.456	0.6487	1.0000
			Gluten intake female	1.1 × 10^−3^	1.4 × 10^−2^	6.6 × 10^−4^	1.604	0.1088	1.0000
GFR (ml/min)	28,677	0.09	Gluten intake	− 8.1 × 10^−2^	− 3.1 × 10^−2^	1.6 × 10^−2^	− 4.987	<0.0001	<0.0001

The results of the multiple linear regression analyses are expressed in terms of the non-standardized coefficient beta (*B*), standardized coefficient beta (*β*), and the adjusted coefficient of determination (R^2^). Holm adjustment was used to control for multiple comparisons. All models were adjusted for sex, age, smoking status, ethnic background, qualifications, total household income per year, energy intake, and physical activity. Since age, sex, and ethnic background are included when calculating GFR, these parameters were removed from the model with GFR as dependent variable. Furthermore, additional covariates were included in models with the following dependent variables. *SBP* and *DBP* percentage body fat, blood pressure medication; *HbA1c* percentage body fat, diabetes status; *Total*, *LDL*, *HDL* *cholesterol*, *triglyceride* percentage body fat, lipid-lowering medication, fiber intake; *CRP* percentage body fat, lipid-lowering medication; *GFR* percentage body fat, diabetes status. Abbreviations are indicated in Table 1. *SE* standard error

### Gluten intake and further markers of adverse body composition and body weight

Associations between gluten intake on the one hand and WC, WHR, WHtR, and BMI on the other hand were elucidated and all *p* values were adjusted for multiple comparisons (Table 2 and Online Resource 6). After adjusting for confounders, gluten intake was associated in females only with WC (*β* = 0.041, *p* < 0.0001; Table 2 and Online Resource 6a), WHtR (*β* = 0.040, *p* < 0.0001; Table 2 and Online Resource 6c), and BMI (*β* = 0.043, *p* < 0.0001; Table 2 and Online Resource 6d). In contrast, gluten intake was not associated with WHR (Table 2 and Online Resource 6b). In female participants, an increase of gluten intake by 1 g/day was related to an increase of WC by 0.09 cm, WHtR by 0.0005, and BMI by 0.04 kg/m^2^ in multiple regression models assuming non-transformed dependent variables (data not shown).

### Gluten intake and metabolic health

**Fig. 2:**
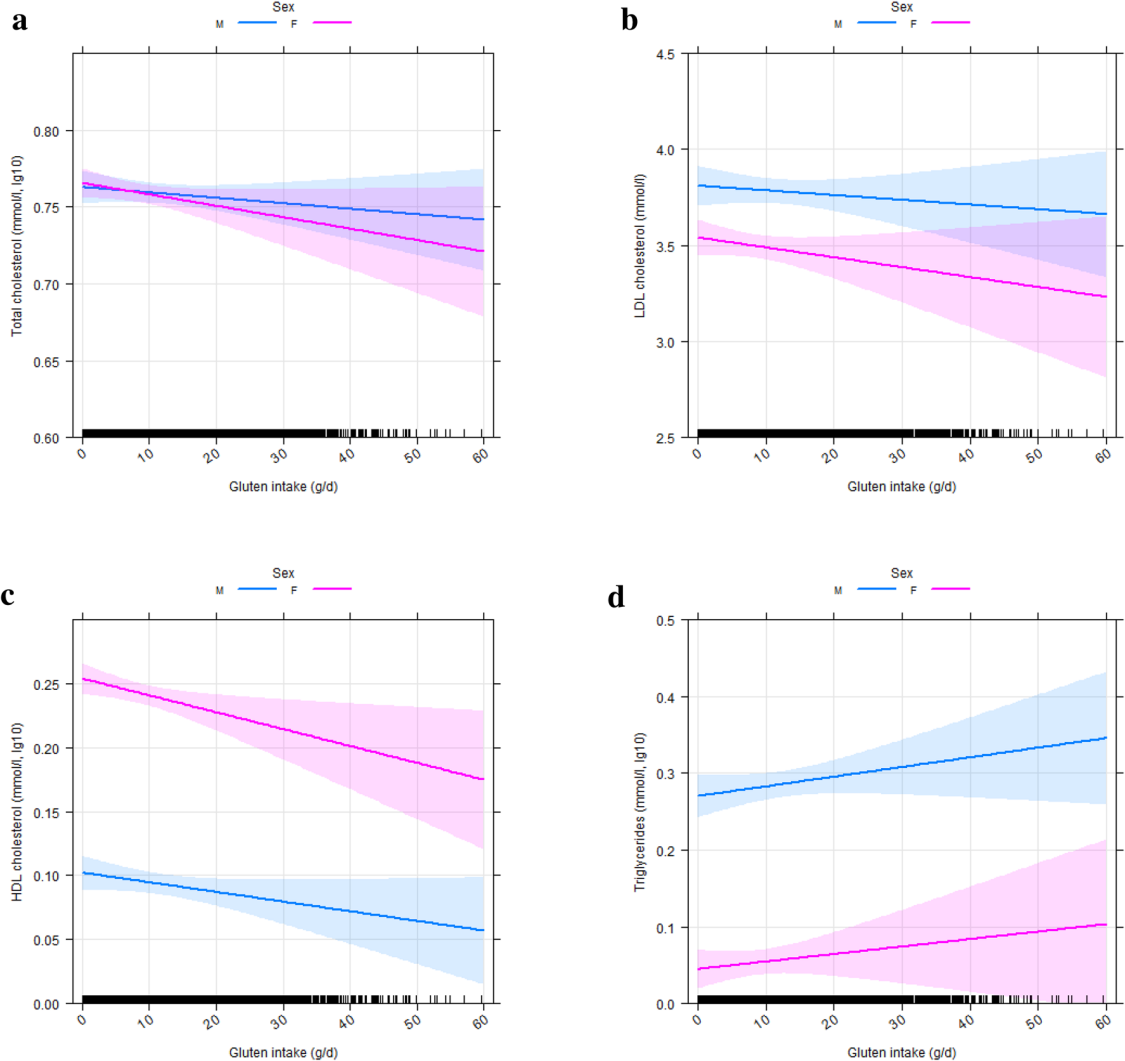
Predictor effects plots of multiple linear regression analysis determining association of serum lipids (dependent variables), i.e. **a** total cholesterol, **b** LDL cholesterol, **c** HDL cholesterol, and **d** triglycerides with gluten intake (independent variable) in models adjusted as summarized in Table 2

Association between gluten intake and metabolic health, i.e. markers of hypertension (SBP, DBP), impaired glucose control (HbA1c), dyslipidemia [total cholesterol, low-density lipoprotein (LDL) cholesterol, high-density lipoprotein (HDL) cholesterol, triglycerides], subclinical inflammation [C-reactive protein (CRP)], and renal complications (GFR) was assessed and all *p* values were adjusted for multiple comparisons (Table 2). Gluten intake was not related to blood pressure and HbA1c in both sexes (Table 2 and Online Resource 7a–c). In contrast, gluten intake was negatively associated with total cholesterol (male: *β* = − 0.031, *p* = 0.0154; female: *β* = − 0.050, *p* < 0.0001) and HDL cholesterol (male: *β* = − 0.052, *p* < 0.0001; female: *β* = − 0.068, *p* < 0.0001) in both sexes, as well as with LDL cholesterol in female subjects (*β* = − 0.035, *p* = 0.0011) (Table 2 and Fig. 2a–c). Gluten intake was positively related to serum triglycerides in males (*β* = 0.043, *p* = 0.0001; Table 2 and Fig. 2d). An increase of gluten intake by 1 g/day was statistically associated with decreases in total, LDL, and HDL cholesterol by 0.006 mmol/l, 0.004 mmol/l, and 0.003 mmol/l, respectively, and an increase in triglycerides by 0.004 mmol/l in multiple regression models including non-transformed dependent variables and no interaction between sex and gluten intake (data not shown). Gluten intake was not a predictor of the inflammatory marker CRP in both sexes (Table 2, Online Resource 7d). Gluten intake was negatively related to GFR after adjustment for confounders (*β* = − 0.031, *p* < 0.0001; Table 2 and Online Resource 7e). An increase in gluten intake by 1 g/day was associated with a decrease in GFR by 0.08 ml/min (Table 2).

### GFD and percentage body fat

Within our UK Biobank cohort, 628 participants (male: 134; female: 494) without history of CD or malabsorption indicated that they were on a GFD. When GFD instead of gluten intake was included in the multiple regression model, percentage body fat was 1.7 (*β* = − 0.022, *p* < 0.0001) percentage points lower in female and 1.1 (*β* = − 0.008, *p* = 0.0527) percentage points lower in male participants on a GFD as compared to subjects on a normal diet (Online Resource 8).

### Predictors of gluten intake

A multiple linear regression analysis with gluten intake as dependent variable was used to examine the association between gluten intake on one hand and sociodemographic characteristics including sex, age, ethnic background, qualifications, and average total household income, as well as lifestyle risk factors including smoking status, energy intake, and physical activity, on the other hand (Online Resource 9). Among the covariates studied, negative predictors of gluten intake were female gender, increasing age, and increasing total household income whereas energy intake was a positive determinant (Online Resource 9).

## Discussion

In the current study, the association between daily gluten intake and percentage body fat as predefined primary objective, as well as WHR and WHtR as markers of body fat distribution (secondary objectives), are elucidated for the first time in human subjects without CD. We showed that dietary gluten intake is associated with percentage body fat after adjusting for confounders. Interestingly, the direction is sex dependent with males showing a negative and females a positive relationship between gluten intake and percentage body fat.

However, this association is not clinically relevant. Thus, an increase of gluten intake by 1 g/day equivalent to half a slice of bread or half a doughnut is related to a decrease in percentage body fat by only 0.03 percentage points in males and an increase in percentage body fat by only 0.04 percentage points in females. In addition, dietary gluten intake predicts WC, WHtR, and BMI but not WHR in female participants of the UK Biobank cohort. Again, these associations are clinically non-relevant with an increase of gluten intake by 1 g/day being related to an increase of WC by 0.09 cm, WHtR by 0.0005, and BMI by 0.04 kg/m^2^.

Previous studies assessing the link between gluten intake and other measures of obesity including body weight, BMI, and WC have yielded conflicting results. In 1095 young healthy adults aged 20 to 29 years, WC is not significantly different between tertiles of gluten intake and no dose–response relationship exists for BMI [25]. A randomized, controlled, crossover trial compares 8-week interventions of low-versus high-gluten diet in 60 middle-aged, healthy adults [41]. At the end of the intervention, low-gluten dieting results in a moderate but significant weight loss (− 0.81 kg, *p* = 0.01) but has no effect on WC (+ 0.14 cm, *p* = 0.86) as compared to the high-gluten diet [41]. In contrast, WC reduction is significantly higher in 23 subjects on a GFD as compared to 22 participants on a control diet while no significant decrease in body weight is observed [42]. In a randomized crossover study comprising 20 hyperlipidemic men and women, an isoenergetic, high-protein diet high in wheat gluten over 1 month improves lipids but does not show effects on body weight as compared to a control diet [43]. Furthermore, protein intake is not related to body fat in tightly controlled feeding studies [44]. In addition, the divergent associations between gluten intake and percentage body fat depending on sex in the present study essentially violate Hill’s criteria for causality [45]. Taking these results and our current findings into consideration, gluten intake does not appear to be an important predictor of adverse body composition related to metabolic disease.

Animal studies suggest that dietary gluten may be beneficial in blood pressure control by inhibiting angiotensin-I-converting enzyme [46, 47]. In the current study, gluten intake is not associated with blood pressure in both sexes. Our results are in line with various epidemiological and intervention studies which also do not find any association between gluten intake and hypertension [25, 42, 43, 48]. Therefore, it is unlikely that gluten intake contributes to blood pressure control in human subjects.

In agreement with our HbA1c results, HbA1c is not different comparing 8 weeks of low-gluten versus high-gluten diet [41]. Furthermore, mean HbA1c is not significantly different between healthy people avoiding gluten and the general population in NHANES [48].

The current study suggests that gluten intake is a negative predictor of total and HDL cholesterol in both sexes and LDL cholesterol in females. Similar to percentage body fat, these associations are not clinically meaningful with an increase of gluten intake by 1 g/day being statistically associated with decreases in total, LDL, and HDL cholesterol by 0.006 mmol/l, 0.004 mmol/l, and 0.003 mmol/l, respectively. Consistent with our findings, HDL cholesterol is significantly higher in GFD as compared to non-GFD participants whereas no difference is seen for total cholesterol in cross-sectional NHANES data [48]. In addition, no significant difference in cholesterol levels is observed in tertiles of gluten intake in another cross-sectional study of young healthy adults [25]. Furthermore, various intervention studies do not show significant associations between gluten intake and cholesterol metabolism [41–43]. In a study from the 1960s, a gluten intake of 100 g/day has cholesterol-lowering effects possibly mediated via increased lipid excretion [49]. However, the gluten dose used in this intervention study is exceedingly high, i.e. more than ten times higher as compared to median intake in our current study. The median gluten intake of UK Biobank participants of 9.7 g/day is well in accordance with published findings in other cohorts which estimate gluten intake in the range from 5 to 13 g/day [17, 18, 50, 51]. In the current report, gluten intake is weakly and positively associated with serum triglycerides in male UK Biobank participants. Previous studies concerning serum triglycerides are contradictory [25, 41–43]. Our results support the assumption that dietary gluten does not adversely affect lipid status in subjects without CD.

Dietary gluten intake is not related to the subclinical inflammation marker CRP in the current study. In agreement with our findings, CRP is not different between tertiles of energy-adjusted gluten intake in healthy young adults [25]. Additionally, CRP, as well as further inflammatory markers, i.e. interleukin-6 and TNFα, is not affected by the extent of gluten intake in a crossover intervention study [41].

To the best of our knowledge, only one study so far has assessed the impact of gluten intake on renal function with short-term high gluten intake not significantly affecting creatinine clearance [43]. However, our analyses show that gluten intake is a negative predictor of GFR. Again, this association is not clinically meaningful with an increase in gluten intake by 1 g/day being associated with a decrease in GFR by 0.08 ml/min.

We further assessed the association between percentage body fat and GFD. In multiple regression analysis, percentage body fat is lower by 1.7 percentage points in females on a GFD. In male participants following a GFD, percentage body fat is 1.1 percentage points less; however, this association does not reach statistical significance. Similar to our findings, NHANES data suggest that healthy people on a GFD have a significantly lower WC as compared to the general population and such a trend (*p* = 0.053) also exists for lower BMI [48]. Nonetheless, it needs to be considered that only a very small subgroup of participants is on a GFD in both studies, i.e. 628 out of 39,927 subjects in the current study and 155 out of 13,523 in NHANES [48].

Taking our findings and published evidence into consideration, gluten intake is not associated with markers of impaired metabolic health in a clinically relevant manner after adjusting for confounding factors. Impaired metabolic health might rather be the consequence of an unbalanced diet in both gluten-containing or gluten-free diets [10]. It needs to be pointed out that our results do not necessarily apply to a population with a larger gluten intake.

Strengths of our study include a large and well-characterized population of almost 40,000 participants, as well as a broad set of clinical and biochemical markers which enable some analyses for the first time. A major limitation of the study is its cross-sectional design which precludes definition of causal relationships. Furthermore, non-fasting blood samples are analyzed which might affect some findings, e.g., on lipid status. However, recent systematic studies have suggested that the difference between fasting and non-fasting samples is small for most lipid parameters [52]. Moreover, estimation of energy and gluten intake relies on a memory-based food recall instrument which might lack reliability leading to under- and over-reporting [53, 54]. To address this limitation, participants with outlier values including discrepancy between basal metabolic rate and energy intake have been excluded from the current analysis. However, it cannot be ruled out that the underlying results from multiple linear regression analyses are due to differences in caloric intake despite the fact that all models have been adjusted for energy intake. In addition, dietary information from only one Oxford WebQ was available at the baseline assessment for each participant.

Despite these points, the current study indicates that gluten intake is not a major contributor to metabolic health in subjects without CD. Therefore, measures to decrease gluten intake are unlikely to provide health benefits for a population in total. Our data further underscore the need to go away from the notion of statistical significance and focus on effect sizes. Our findings support the concept that adiposity and metabolic disease are driven by factors other than gluten intake. Further prospective studies on gluten intake in relation to morbidity and mortality are necessary to provide even more definitive conclusions.

## Electronic supplementary material

Below is the link to the electronic supplementary material.

Supplementary file1 (PDF 429 kb)

## Data Availability

Restrictions apply since UK Biobank data are not publicly available and were used under license for application 47144. However, data are available from the authors upon reasonable request and with UK Biobank permission.

## Code availability

Data were analyzed using R software version 3.6.1**.** Statistical code generating the results of this manuscript can be requested from the authors.
