# Supplementary material for: Gluten intake and metabolic health: conflicting findings from the UK Biobank
Source: Eur J Nutr. 2020 Aug 6;60(3):1547–59. doi: 10.1007/s00394-020-02351-9 (PMC7987594; doi:10.1007/s00394-020-02351-9)
Supplement: Supplementary file 1 — Supplementary file1 (PDF 429 kb) [file 394_2020_2351_MOESM1_ESM.pdf]

Supplementary Material

European Journal of Nutrition

Gluten intake and metabolic health: conflicting findings from the UK Biobank

Authors: Inken Behrendt[1*]; Mathias Fasshauer[1,2,3#]; Gerrit Eichner[4#]

1. Institute of Nutritional Science, Justus-Liebig University of Giessen, Giessen, Germany.
2. Department of Internal Medicine (Endocrinology, Nephrology, and Rheumatology), University of Leipzig, Leipzig, Germany.
3. Leipzig University Medical Center, IFB AdiposityDiseases, Leipzig, Germany.
4. Mathematical Institute, Justus-Liebig University of Giessen, Giessen, Germany.

#These authors contributed equally to this work.
*Corresponding author. Mailing address: Goethestr. 55, 35390 Giessen, Germany. Phone: +49 641 9939067. Fax: +49 641 9939069. E-mail: inken.behrendt@ernaehrung.uni-giessen.de.

Supplemental Figure legends

Online Resource 1
Venn diagram depicting number of participants excluded by six exclusion criteria

Online Resource 2
Boxplots depicting **a** gluten intake and **b** energy-adjusted gluten intake depending on sex

Online Resource 3
Predictor effects plots of multiple linear regression analysis determining association of percentage body fat (dependent variable) with **a** energy-adjusted gluten intake, **b** sex, **c** age, **d** smoking status, **e** qualifications, **f** ethnic background, **g** income, and **h** MET per week (independent variables)

Online Resource 4
Predictor effects plots of multiple linear regression analysis determining association of percentage body fat (dependent variable) with **a**, **b** gluten intake adjusted as summarized in Fig. 1 or **c**, **d** energy-adjusted gluten intake adjusted as summarized in Online Resource 3 (independent variables) depending on sex. Models in **a**, **c** are further adjusted for markers of diet quality, i.e. consumption of cooked vegetables, salad, fresh fruit, oily fish, processed meat, added salt, saturated fat, polyunsaturated fat, and fiber. Models in **b**, **d** include participants with implausible energy intake

Online Resource 5
Predictor effects plots of multiple linear regression analysis determining association of percentage body fat (dependent variable) with **a-c** gluten intake adjusted as summarized in Fig. 1 or **d-f** energy-adjusted gluten intake adjusted as summarized in Online Resource 3 (independent variables) depending on sex. Participants with **a**, **d** diabetes mellitus (i.e. at least one of the following criteria: established diagnosis of diabetes mellitus, diabetes medication, HbA1c ≥ 48 mmol/mol Hb, non-fasting glucose ≥ 11.1 mmol/l), **b**, **e** arterial hypertension (i.e. at least one of the following criteria: blood pressure medication, SBP > 140 mmHg, DBP > 90 mmHg), or **c**, **f** dyslipidemia (i.e. at least one of the following criteria: cholesterol medication, HDL cholesterol ≤ 1.03 mmol/l for male or ≤1.29 mmol/l for female) are excluded from the analysis

Online Resource 6
Predictor effects plots of multiple linear regression analysis determining association of further markers of obesity (dependent variables), i.e. **a** WC, **b** WHR, **c** WHtR, and **d** BMI with gluten intake (independent variable) in models adjusted as summarized in Table 2

Online Resource 7
Predictor effects plots of multiple linear regression analysis determining association of markers of metabolic health (dependent variables), i.e. **a** SBP, **b** DBP, **c** HbA1c, **d** CRP, and **e** GFR with gluten intake (independent variable) in models adjusted as summarized in Table 2

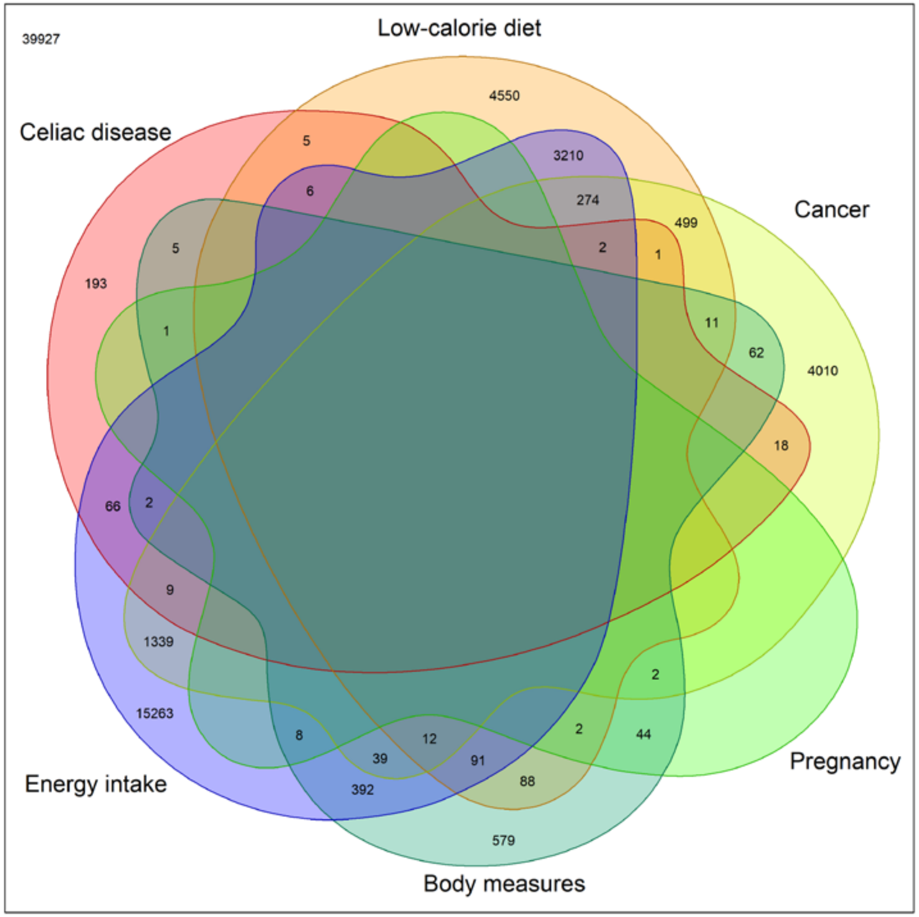

Online Resource 1

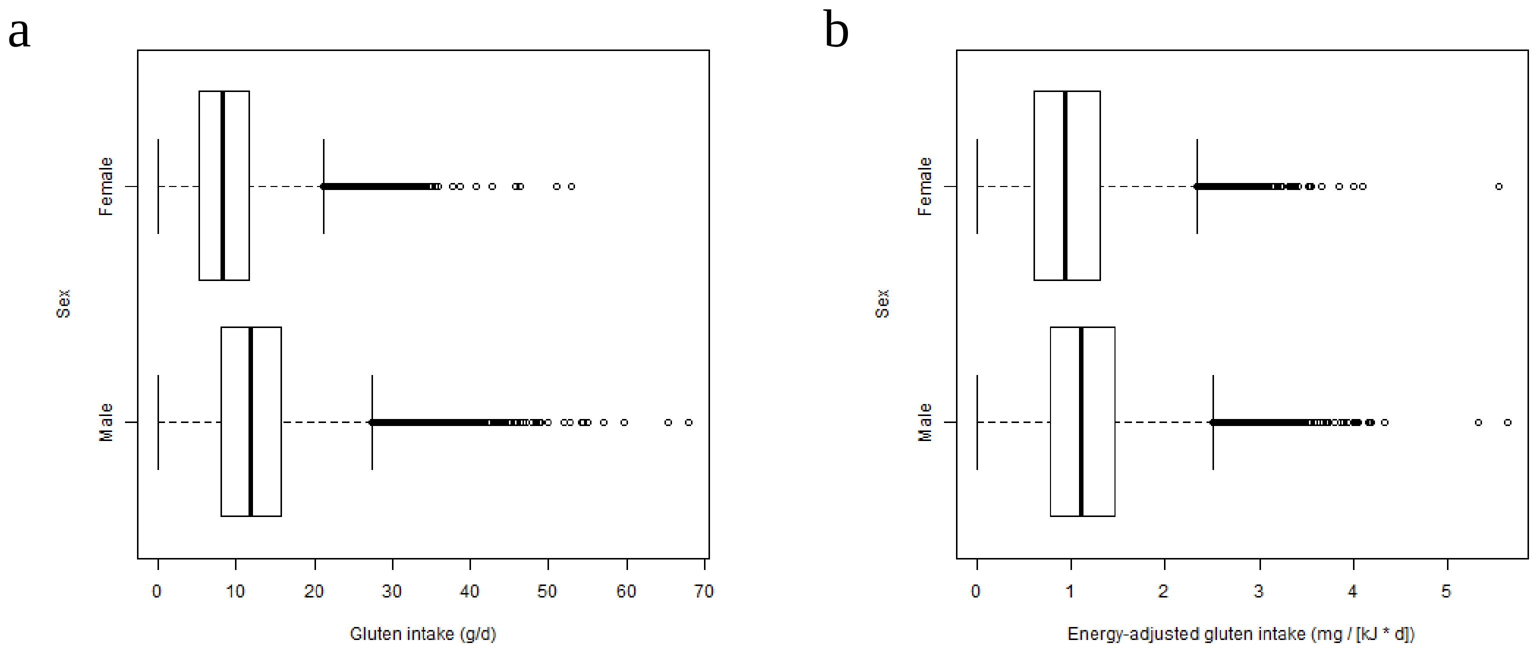

Online Resource 2

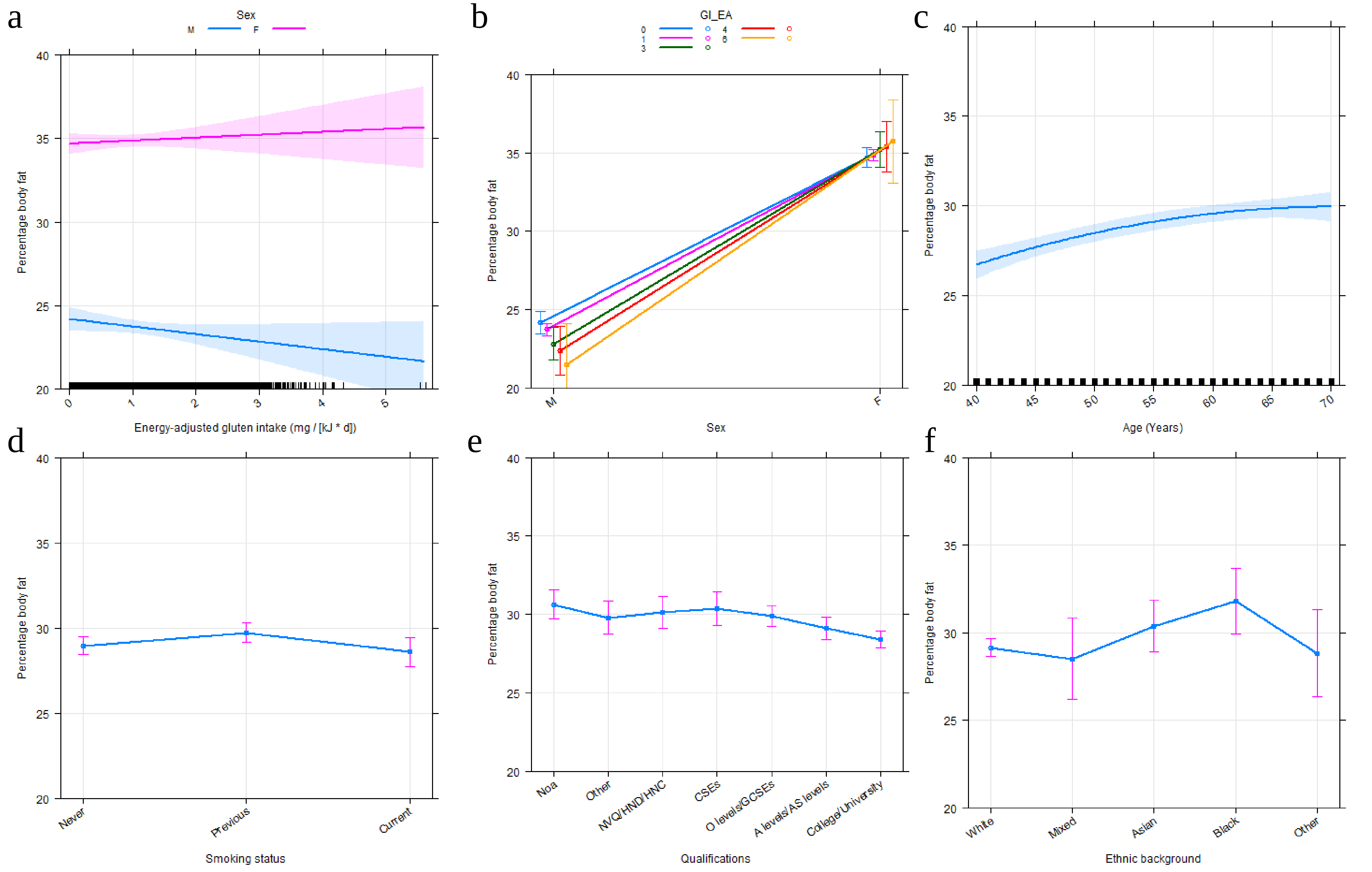

Online Resource 3

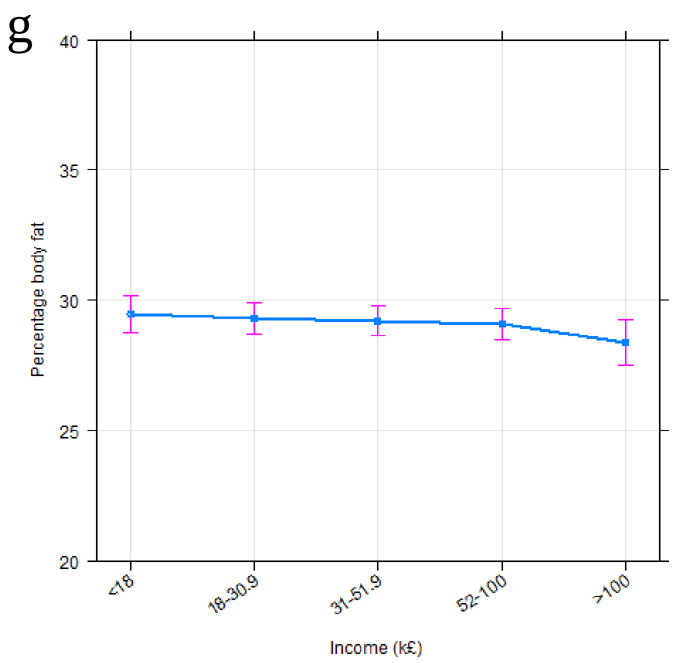

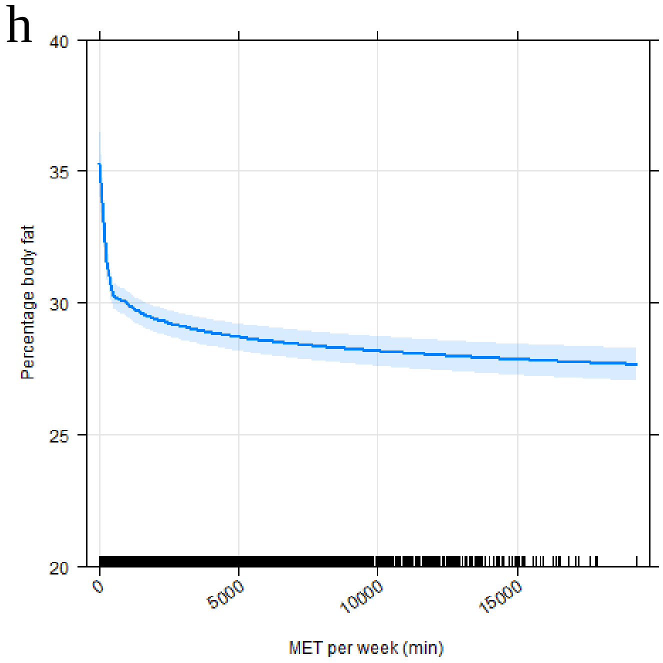

Online Resource 3

Adjustment for markers of diet quality | **Inclusion of participants with implausible energy intake**

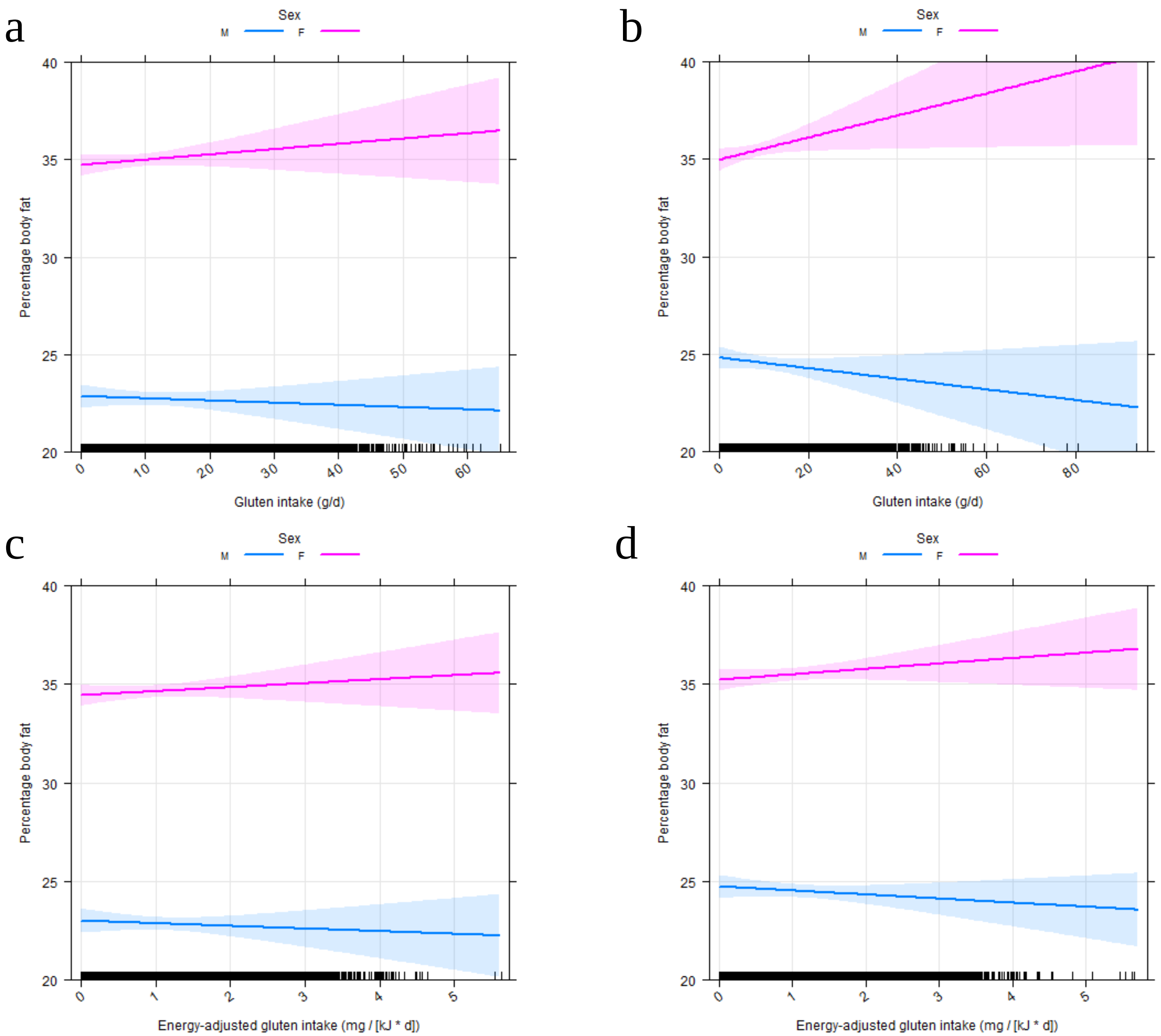

Online Resource 4

Exclusion of participants with diabetes mellitus | **Exclusion of participants with arterial hypertension** | **Exclusion of participants with dyslipidemia**

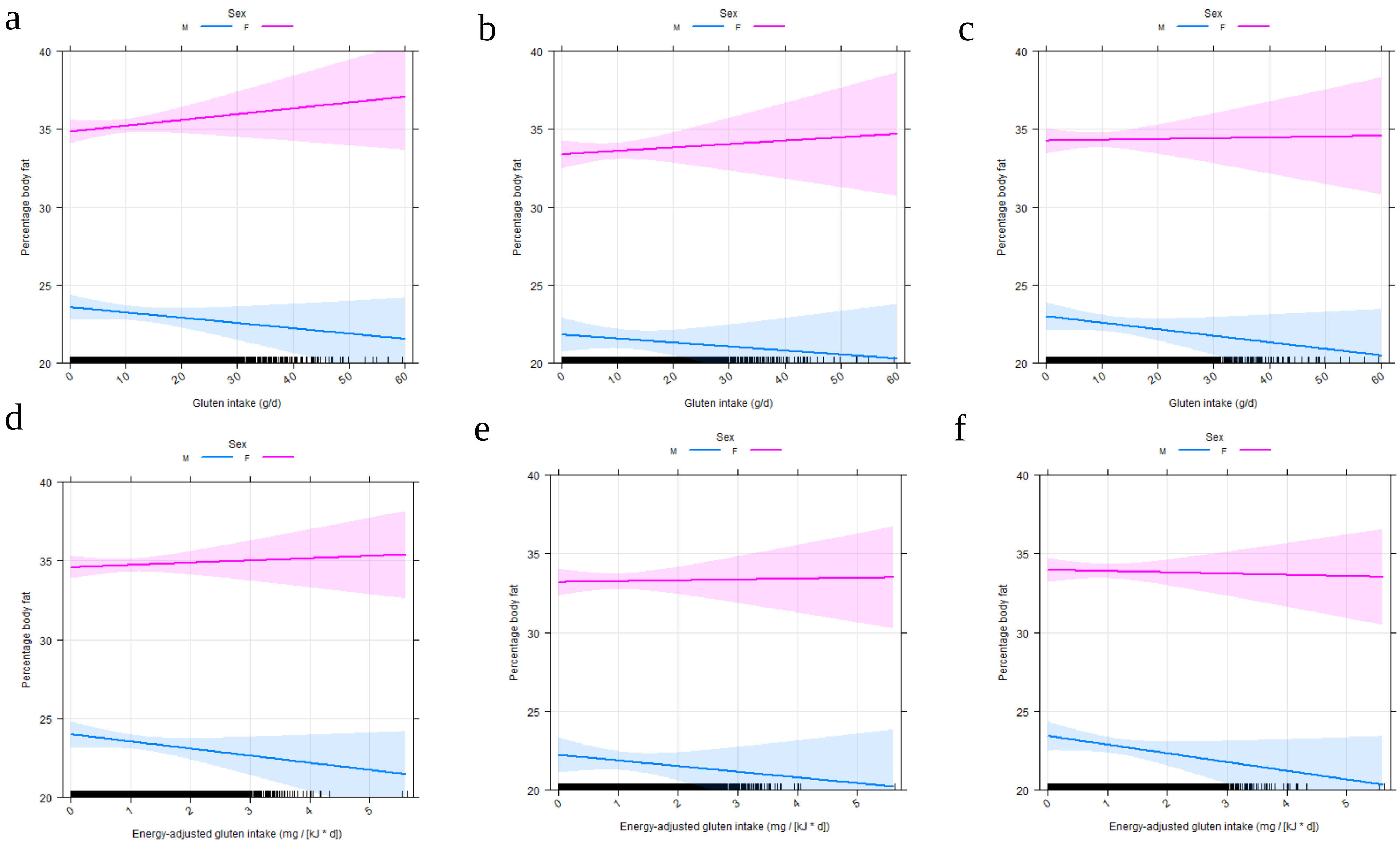

Online Resource 5

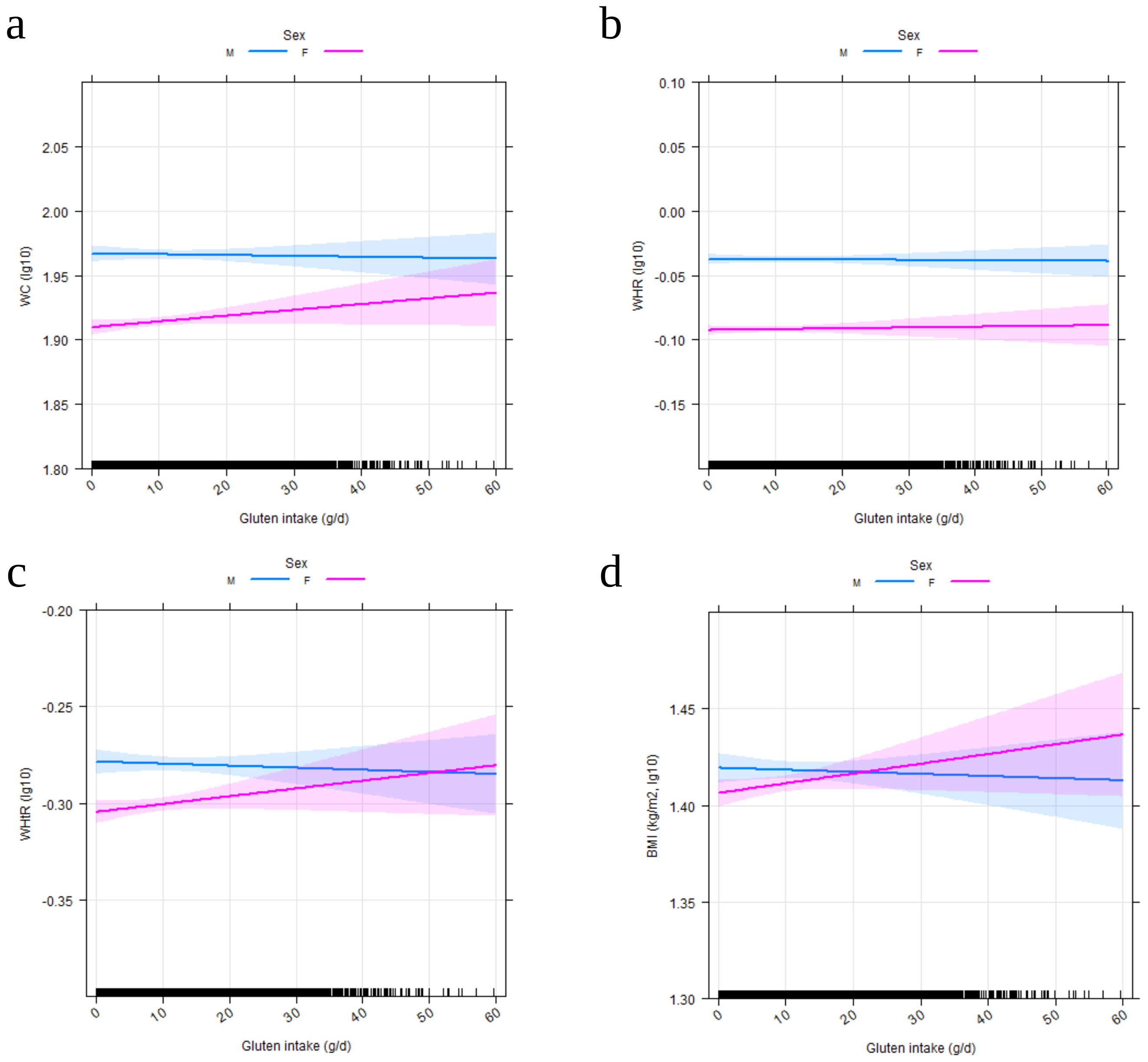

Online Resource 6

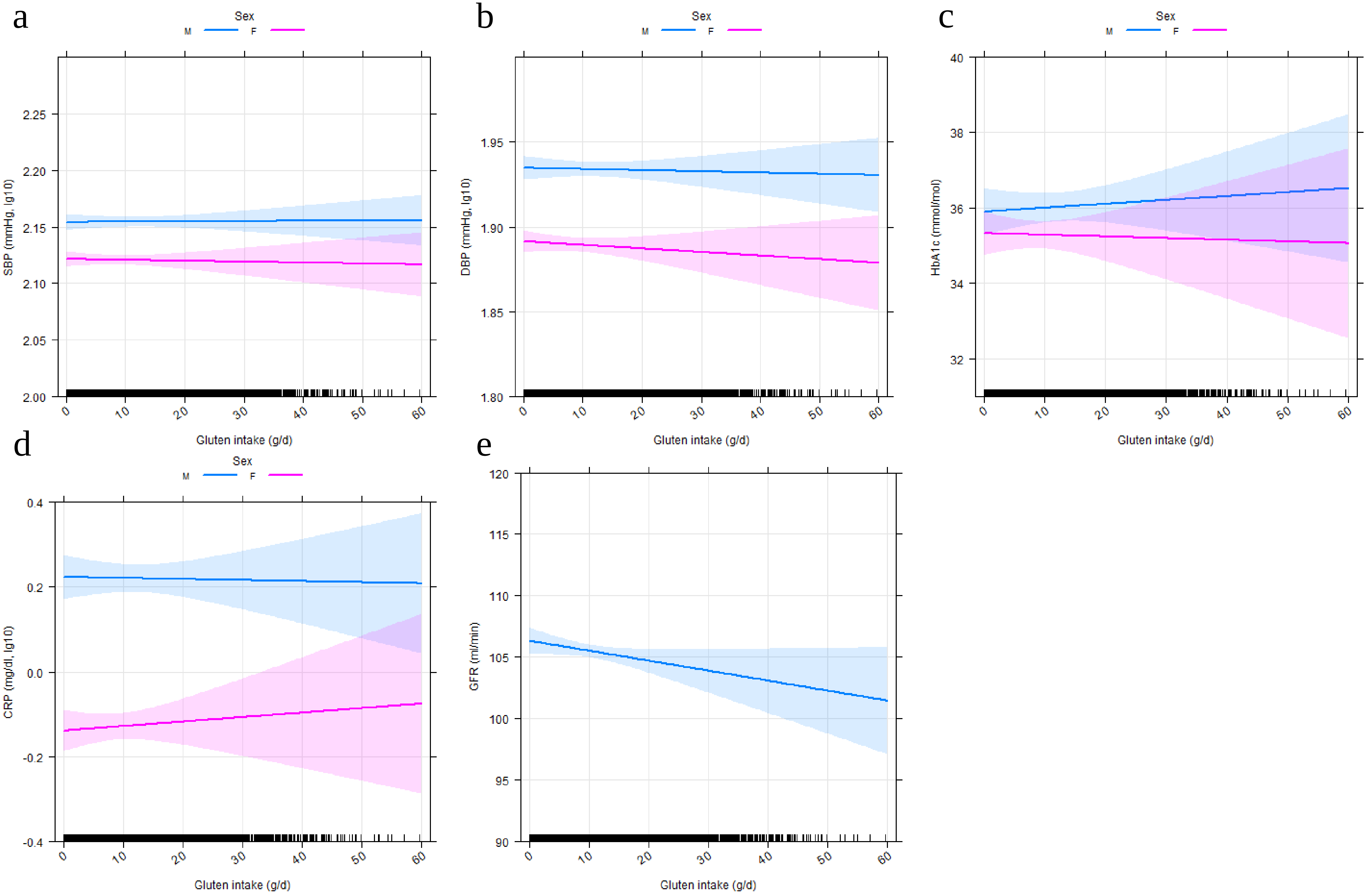

Online Resource 7

Online Resource 8

Multiple linear regression analysis using percentage body fat as the dependent variable and gluten-free diet instead of gluten intake as independent variable (*n*=30,460)

**Independent variable**	***B***	***β***	**SE**	***t* value**	***p* value**
Intercept	-2.1 x 10^1		2.2 x 10^0	-9.544	<0.0001
Gluten-free diet (reference: No GFD)					
- Yes Male	-1.1 x 10^0	-7.9 x 10^{-3}	5.7 x 10^{-1}	-1.938	0.0527
- Yes Female	-1.7 x 10^0	-2.2 x 10^{-2}	3.1 x 10^{-1}	-5.475	<0.0001
Sex (reference: Male)					
- Female	1.2 x 10^1	7.2 x 10^{-1}	7.6 x 10^{-2}	157.366	<0.0001
Age	4.5 x 10^{-1}	4.4 x 10^{-1}	6.0 x 10^{-2}	7.464	<0.0001
Age^2	-3.0 x 10^{-3}	-3.2 x 10^{-1}	5.5 x 10^{-4}	-5.386	<0.0001
Smoking status (reference: Never)					
- Previous	7.4 x 10^{-1}	4.2 x 10^{-2}	7.4 x 10^{-2}	9.947	<0.0001
- Current	-4.0 x 10^{-1}	-1.3 x 10^{-2}	1.3 x 10^{-1}	-3.126	0.0018
Ethnic background (reference: White)					
- Black	2.6 x 10^0	3.5 x 10^{-2}	3.0 x 10^{-1}	8.564	<0.0001
- Asian	1.4 x 10^0	2.4 x 10^{-2}	2.4 x 10^{-1}	5.826	<0.0001
- Mixed	-5.5 x 10^{-1}	-5.8 x 10^{-3}	3.8 x 10^{-1}	-1.422	0.1551
- Other	-4.4 x 10^{-1}	-4.3 x 10^{-3}	4.1 x 10^{-1}	-1.067	0.2859
Qualifications (reference: Noa)					
- Other	-8.4 x 10^{-1}	-2.1 x 10^{-2}	2.1 x 10^{-1}	-4.110	<0.0001
- NVQ or HND or HNC equivalent	-4.5 x 10^{-1}	-1.2 x 10^{-2}	2.0 x 10^{-1}	-2.269	0.0233
- CSEs or equivalent	-2.6 x 10^{-1}	-6.4 x 10^{-3}	2.1 x 10^{-1}	-1.234	0.2172
- O levels/GCSEs or equivalent	-6.9 x 10^{-1}	-3.4 x 10^{-2}	1.5 x 10^{-1}	-4.569	<0.0001
- A levels/AS levels or equivalent	-1.5 x 10^0	-6.2 x 10^{-2}	1.6 x 10^{-1}	-9.248	<0.0001
- College or university degree	-2.2 x 10^0	-1.3 x 10^{-1}	1.5 x 10^{-1}	-15.038	<0.0001
Total household income per year (£; reference: <18000)					
- 18000-30999	-1.4 x 10^{-1}	-7.0 x 10^{-3}	1.1 x 10^{-1}	-1.231	0.2184
- 31000-51999	-1.9 x 10^{-1}	-1.0 x 10^{-2}	1.1 x 10^{-1}	-1.684	0.0921
- 52000-100000	-2.8 x 10^{-1}	-1.5 x 10^{-2}	1.2 x 10^{-1}	-2.390	0.0169
- >100000	-9.8 x 10^{-1}	-3.2 x 10^{-2}	1.6 x 10^{-1}	-6.218	<0.0001
Lg_{10} Energy intake	9.0 x 10^0	1.1 x 10^{-1}	3.6 x 10^{-1}	24.973	<0.0001
Lg_{10} MET per week	-1.8 x 10^0	-1.3 x 10^{-2}	5.8 x 10^{-2}	-30.757	<0.0001

The results of the multiple linear regression analyses are expressed in terms of the non-standardized coefficient beta (*B*), standardized coefficient beta (*β*), and the adjusted coefficient of determination (R^2). Adjusted R^2=0.50, *p* overall model: <0.0001. Abbreviations are indicated in table 1. *SE* standard error.

Online Resource 9

Multiple linear regression analysis using gluten intake as the dependent variable (*n*=30,460)

**Independent variable**	***B***	***β***	***SE***	***t* value**	***p* value**
Intercept	-6.0×10^{1}		1.4×10^{0}	-43.343	<0.0001
Sex (reference: Male)					
- Female	-2.1×10^{0}	-1.8×10^{-1}	6.9×10^{-2}	-31.244	<0.0001
Age	-6.1×10^{-2}	-8.4×10^{-2}	4.1×10^{-3}	-14.686	<0.0001
Smoking status (reference: Never)					
- Previous	-3.5×10^{-1}	-2.8×10^{-2}	6.7×10^{-2}	-5.241	<0.0001
- Current	-3.2×10^{-1}	-1.5×10^{-2}	1.2×10^{-1}	-2.788	0.0053
Ethnic background (reference: White)					
- Black	-1.3×10^{0}	-2.4×10^{-2}	2.7×10^{-1}	-4.657	<0.0001
- Asian	4.2×10^{-1}	1.0×10^{-2}	2.2×10^{-1}	1.960	0.0500
- Mixed	-9.8×10^{-1}	-1.4×10^{-2}	3.5×10^{-1}	-2.814	0.0049
- Other	4.0×10^{-2}	5.5×10^{-4}	3.7×10^{-1}	0.107	0.9151
Qualifications (reference: Noa)					
- Other	-5.1×10^{-1}	-1.8×10^{-2}	1.9×10^{-1}	-2.719	0.0066
- NVQ or HND or HNC equivalent	-1.6×10^{-1}	-5.7×10^{-3}	1.8×10^{-1}	-0.856	0.3922
- CSEs or equivalent	-1.5×10^{-1}	-5.1×10^{-3}	1.9×10^{-1}	-0.792	0.4287
- O levels/GCSEs or equivalent	-4.7×10^{-1}	-3.1×10^{-2}	1.4×10^{-1}	-3.394	0.0007
- A levels/AS levels or equivalent	-6.4×10^{-1}	-3.7×10^{-2}	1.5×10^{-1}	-4.385	<0.0001
- College or university degree	-5.2×10^{-1}	-4.3×10^{-2}	1.3×10^{-1}	-3.918	<0.0001
Total household income per year (£; reference: <18000)					
- 18000-30999	-2.9×10^{-1}	-2.1×10^{-2}	1.0×10^{-1}	-2.915	0.0036
- 31000-51999	-6.7×10^{-1}	-5.0×10^{-2}	1.0×10^{-1}	-6.636	<0.0001
- 52000-100000	-9.0×10^{-1}	-6.4×10^{-2}	1.1×10^{-1}	-8.364	<0.0001
- >100000	-1.5×10^{0}	-6.7×10^{-2}	1.4×10^{-1}	-10.458	<0.0001
Lg_{10} Energy intake	1.9×10^{1}	3.3×10^{-1}	3.3×10^{-1}	58.904	<0.0001
Lg_{10} MET per week	-8.4×10^{-2}	-8.2×10^{-3}	5.3×10^{-2}	-1.583	0.1135

The results of the multiple linear regression analyses are expressed in terms of the non-standardized coefficient beta (*B*), standardized coefficient beta (*β*), and the adjusted coefficient of determination (R^2). Adjusted R^2=0.20, *p* overall model: <0.0001. Abbreviations are indicated in table 1. *SE* standard error.
